# Operation rate and cancer prevalence among thyroid nodules with FNAC report of suspicious for malignancy (TIR4) or malignant (TIR5) according to Italian classification system: a systematic review and meta-analysis

**DOI:** 10.1007/s12020-022-03165-x

**Published:** 2022-08-20

**Authors:** Pierpaolo Trimboli, Giulia Ferrarazzo, Arnoldo Piccardo, Barbara Lucchini, Cosimo Durante

**Affiliations:** 1 Servizio di Endocrinologia e Diabetologia, Ente Ospedaliero Cantonale (EOC), Bellinzona, Switzerland; 2 Facoltà di Scienze Biomediche, Università della Svizzera Italiana (USI), Lugano, Switzerland; 3 Medicina Nucleare, Ospedale Villa Scassi Hospital, Genoa, Italy; 4 Struttura Complessa di Medicina Nucleare, E.O. Ospedali Galliera, Genoa, Italy; 5 Department of Translational and Precision Medicine, “Sapienza” University of Rome, Rome, Italy

**Keywords:** Thyroid, Fine-needle aspiration (FNA), Nodules, Carcinoma, Risk of malignancy

## Abstract

**Background:**

In the Italian system for reporting thyroid cytology (ICCRTC), nodules suspicious for (TIR4) and consistent with (TIR5) malignancy are thought being 5% and 4–8% of all biopsies and having risk of malignancy of 60–80% and >95%, respectively. However, no evidence-based data exist about these figures. The present systematic review aimed at achieving solid estimates about TIR4 and TIR5 also considering potential influencing factors.

**Methods:**

The review was conducted according to MOOSE. Databases of Google Scholar and Cochrane were searched. No language restriction was used. The last search was performed on February 26th 2022. Quality assessment was performed. Proportion meta-analyses were performed using random-effect model. Statistical analyses were performed using OpenMeta [Analyst].

**Results:**

The online search retrieved 271 articles and 16 were finally included for quantitative analysis. The risk of bias was generally low. The pooled cancer prevalence in TIR4 was 92.5% (95%CI 89.4–95.6%) with unexplained moderate heterogeneity. The pooled cancer rate among TIR5 was 99.7% (95%CI 99.3–100%) without heterogeneity. The resection rate in TIR4 and TIR5 showed heterogeneity, being the latter explained when using their prevalence among biopsies: the higher the prevalence, the higher the operation rate. The pooled risk difference between TIR5 and TIR4 was significant (OR 11.153).

**Conclusions:**

These figures can form the basis for the next updated version of ICCRTC. Any institution using ICCRTC should revise its series of TIR4/TIR5 to calculate the cancer rate, and, importantly, consider the modifiers of the risk of malignancy. A cross check among institutions is advised.

## Introduction

Thyroid nodule (TN) is a largely diffused and often incidentally discovered pathological entity. Since the vast majority of TNs is benign, the first aim in clinical practice is to exclude cancer, and ultrasound (US)-guided fine-needle aspiration cytology (FNAC) is pivotal in this context [1, 2]. In fact, with the exception of indeterminate and inconclusive cases accounting as a whole for 20–30% of all FNACs, cytological examination can accurately discriminate samples without cancer features and consistent with benign lesions from specimens consistent with or suspicious for malignancy. On the International scene, two major guidelines for reporting and classification of TN FNAC exist, the UK Royal College of Pathologists (RCPath) [3], and the most widely used system such as The Bethesda System for Reporting Thyroid Cytopathology (TBSRTC) [4]. In these guidelines, TN FNAC samples suspicious for malignancy are classified as Thy 4 and Bethesda V, while those specimens diagnostic for malignancy as Thy 5 and Bethesda VI, respectively. In addition to the above most recognized guidelines [3, 4], the Italian consensus for the classification and reporting of thyroid cytology (ICCRTC) was initially proposed in 2010 [5] and then updated in 2014 [6]. In this Italian proposal, TN FNAC suspicious for malignancy is classified as TIR4 while that consistent with malignancy as TIR5. Because of the high/very high expected risk of malignancy in these two FNAC categories (i.e., 60–80% in TIR4, >95% in TIR5), surgery is always indicated for such patients. Furthermore, it is estimated that the frequency of TIR4 and TIR5 cases among all FNACs accounts for 5% and 4–8%, respectively. These figures were estimated based on sparse data or findings reported in the other guidelines [3, 4]. One single previous meta-analysis exists on this topic [7], only six studies were included, and a small number of cases was pooled, i.e., 589 nodules of which 203 TIR4 and 386 TIR5. The pooled cancer rate was 85% and 99% in TIR4 and TIR5, respectively, with no heterogeneity; however, it is worth noting that the rate of individuals undergoing surgery, a factor with potential to influence the cancer rate, was neither analyzed nor extracted. Since these findings are at variance from that estimated in the ICCRTC guidelines, and their reliability is hampered by the limitations in the data measures, a revision of the literature is warranted to confirm or not these results.

The present systematic review was undertaken to achieve more robust information about FNAC report of TIR4 and TIR5 according to ICCRTC. In particular, the present study aimed to achieve high-evidence estimates of risk of malignancy of these categories, also considering the operation rate and other potential influencing factors.

## Material and Methods

### Conduct and registration of review

The systematic review was conducted according to Meta-analysis Of Observational Studies in Epidemiology (MOOSE) [8].

### Search strategy

To extend to the largest number of publications about ICCRTC, the specific strategy was aimed at retrieving all original studies citing it. The online citation databases of Google Scholar and Cochrane were searched. No language restriction was used. A beginning date limit was 2014 as the date of ICCRCT publication. The last search was performed on February 26^th^ 2022. Reference list of the included articles were also screened to find further studies.

### Study selection

The selection strategy was aimed at including only those studies reporting preoperative and postoperative data of both TIR4 and TIR5 cases to analyze their cancer rate at histology, also considering the operation rate and other potential influencing factors. With this perspective, those studies reporting only TIR4 or TIR5 were not regarded as eligible. Firstly, all records found by the planned strategy were screened and two researchers (GF, PT) independently reviewed titles and abstracts of the retrieved articles and selected those eligible. Secondly, only original papers reporting data of TIR4 and TIR5 were initially included while other type of articles (i.e., review, editorial, letter, comment) were not. After the initial selection, following exclusion criteria were applied: (a) articles not within the field of interest of the review; (b) articles lacking of preoperative or histological data; (c) overlapping studies; (d) pediatric patients; (e) series including less than 10 cases of TIR4 and/or TIR5. Discordances were solved in a final mutual discussion among the authors.

### Data extraction

Following information was extracted independently by two investigators (GF, PT) from all included studies: (1) general study information (authors and their country of origin, year of publication,); (2) enrolment of data of FNACs according to ICCRTC (prospective using ICCRTC during clinical practice or retrospective re-classifying according to ICCRTC all FNACs performed before 2014); (3) total number of FNACs performed during the study period; (4) number of TIR4 and TIR5 during the study period; (5) number of TIR4 and TIR5 operated during the study period; (6) number of cancers among TIR4 and TIR5 operated. Lacking data could be required to corresponding authors of papers, when appropriate. Discordant data were cross-checked, discussed and solved among all authors.

### Study quality assessment

The risk of bias for included studies was assessed by two reviewers (GF, PT) through the National Heart, Lung, and Blood Institute Quality Assessment Tool for Observational Studies [9].

### Statistical analysis

Proportion meta-analyses were performed according to DerSimonian and Laird method (random-effects model) [10] to calculate (1) the risk of cancer among TIR4 and TIR5, and (2) the resection rate among TIR4 and TIR5. A pooled risk difference meta-analysis was performed to calculate the cancer risk difference between TIR4 and TIR5. Forest plots illustrated pooled data with 95% confidence intervals (95% CI). I_2_ index was used to evaluate the inconsistency where <25% means no heterogeneity, 25–50% mild heterogeneity, 50–75% moderate heterogeneity, and >75% high heterogeneity. When heterogeneity was found, it was explored performing meta-regression analysis and/or subgroup analysis. In the latter analysis, a significant difference was assessed when there is a not overlapping 95%CI between two subgroups. Statistical significance was set at *p* = 0.05. Statistical analyses were performed using OpenMeta[Analyst] software (Center for Evidence Synthesis in Health, Brown University, Providence, RI, USA).

## Results

### Eligible articles

**Fig. 1:**
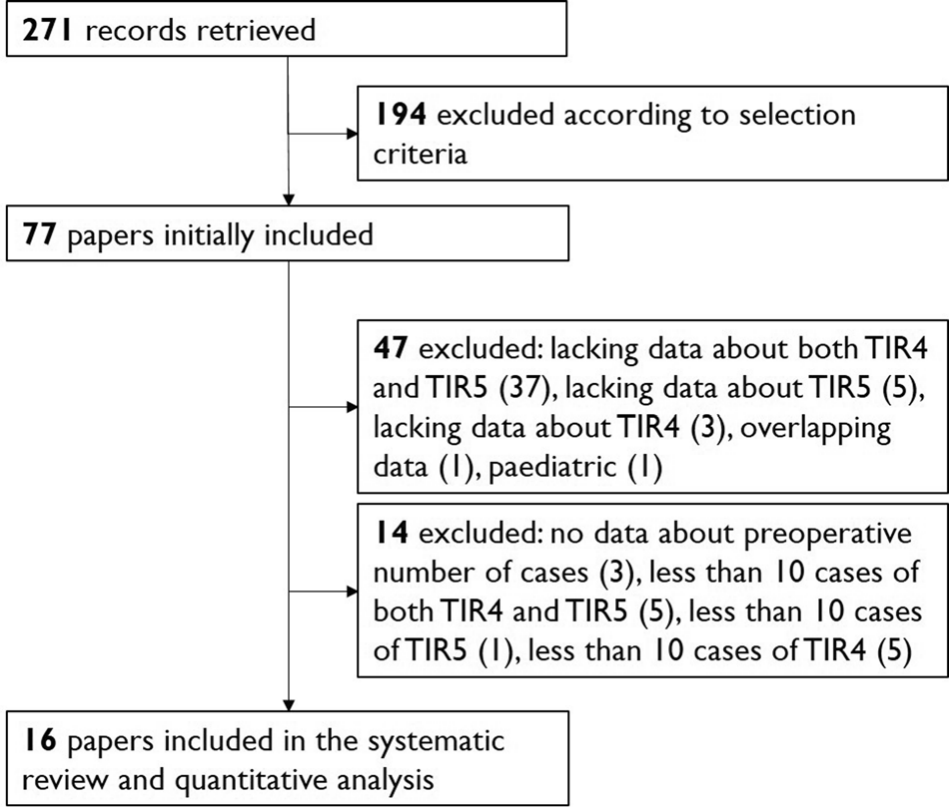
Flow of records found

After excluding duplicate, the online search retrieved 271 articles. Among these, according to selection criteria, 77 were initially selected and, as illustrated in Fig. 1, 16 [11–26] were finally included in the present systematic review and the quantitative analysis.

### Qualitative analysis (systematic review)

The 16 articles were published between 2014 and 2021 in journals in the field of endocrinology (*n* = 8), cytopathology (*n* = 5), oncology (*n* = 2), and biology (*n* = 1). All studies were published by Italian authors and one series was shared with French researchers. Total number of TIR4 and TIR5 was 740 and 1313, respectively. Overall number of FNACs reported in the 16 studies was 21628 and mean frequency of TIR4 and TIR5 among FNACs was 3.4 and 6.1%, respectively. Sample size ranged from 11 to 129 TIR4 and from 20 to 307 TIR5. The largest majority of both TIR4 and TIR5 was operated upon with histological diagnosis. Mean frequency of TIR4 and TIR5 reports among all FNACs was 3.4 and 6.1%, respectively, with a mean ratio TIR5:TIR4 of 1.97:1. Table 1 details general features of the 16 studies.

**Table 1:** Main characteristics of the 16 included studies

First author, year	Ref.	Journal	Country	TIR4			TIR5		
				Tot	Operated	% among FNACs	Tot	Operated	% among FNACs
Pagni, 2014	[11]	Endocrine	Italy	53	53	18.9	71	71	25.3
Bizzarro, 2016	[12]	Cancer Cytopathol	Italy	24	24	19.8	50	50	41.3
Bellevicine, 2016	[13]	Cytopathology	Italy	42	17	1.1	81	54	2.2
Straccia, 2017	[14]	Cytopathology	Italy	99	62	2.4	208	173	5.1
Rezig, 2018	[15]	Metabolomics	France-Italy	15	15	15.3	20	20	20.4
Fish, 2018	[16]	Clin Thyroidol	Italy	11	11	1.8	45	37	7.3
Macerola, 2019	[17]	J Endocrinol Invest	Italy	11	10	1.8	45	38	7.3
Straccia, 2019	[18]	Cytopathology	Italy	20	19	23.3	52	52	60.5
Fulciniti, 2019	[19]	Clin Endocrinol	Italy	18	18	15.7	42	42	36.5
Arena, 2019	[20]	Horm Metab Res	Italy	70	70	17.2	43	43	10.5
Censi, 2019	[21]	Eur J Endocrinol	Italy	129	129	29.6	307	307	70.4
Sponziello, 2020	[22]	Endocrine	Italy	24	24	20.5	32	32	27.4
Dell’Aquila, 2020	[23]	Cancer Cytopathol	Italy	53	53	22.5	58	58	24.6
Giuliano, 2020	[24]	Endocrines	Italy	44	44	11.0	58	58	14.5
Leni, 2021	[25]	Cancers	Italy	12	11	2.5	17	17	3.5
Poma, 2021	[26]	Cancers	Italy	115	115	1.2	184	184	1.9

### Study quality assessment

The risk of bias of included studies is reported in Supplemental Table. Study question, exposure of interest, timeframe, and outcome measures were adequate in all cases. Four issues were not available in all studies. In the remaining issue the overall risk of bias was low.

### Quantitative analysis (meta-analysis)

**Fig. 2:**
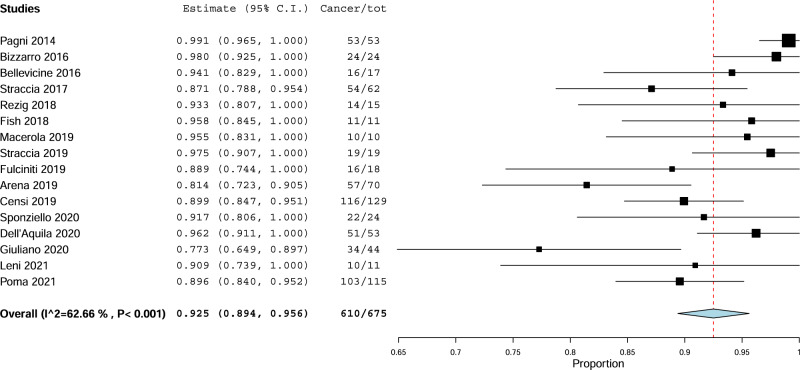
Pooled cancer prevalence among TIR4. Blue diamond indicates the pooled cancer prevalence in the 16 studies and its wideness indicates 95%CI. The cancer rate with 95%CI of any study is illustrated by black square and black line, where square size is according to study sample size

First, the pooled prevalence of cancer among TNs with TIR4 FNAC was evaluated. A 92.5% (95%CI from 89.4 to 95.6%) cancer rate was found with moderate heterogeneity (I_2_ 62%) (Fig. 2). The heterogeneity was explored considering several covariates, such as study sample size (continuous variable), operation rate (continuous variable), percentage of TIR4 among overall series of TNs (continuous variable), and retrospective or prospective design (dichotomic variable), but it remains unsolved. Overall, the pooled operation rate observed in TIR4 TNs was 95.5% (95%CI from 93 to 98%) with high heterogeneity (I_2_ 88%). The latter was explored and then explained when using prevalence of TIR4 among all FNACs as covariate (continuous variable); the higher the prevalence of TIR4 among FNACs, the higher the operation rate (*p* = 0.014).

**Fig. 3:**
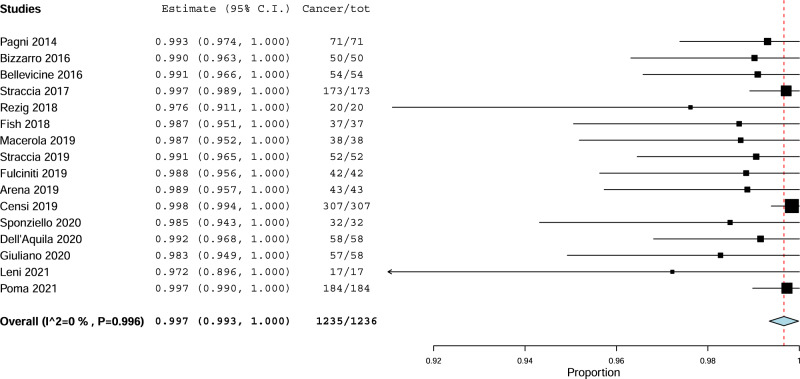
Pooled cancer prevalence among TIR5. Blue diamond indicates the pooled cancer prevalence in the 16 studies and its wideness indicates 95%CI. The cancer rate with 95%CI of any study is illustrated by black square and black line, where square size is according to study sample size

Second, the pooled cancer rate among TIR5 was evaluated. A 99.7% (95%CI from 99.3 to 100%) percentage of cancer rate was found without heterogeneity (I_2_ 0%) (Fig. 3). Overall, only one false positive case among 1236 TIR5 TNs was recorded. The resection rate among TIR5 TNs was 97.3% (95%CI from 95.9 to 98.8%) with high heterogeneity (I_2_ 85%). As for the case of TIR4, the latter was explained when using prevalence of TIR5 among all FNACs as covariate (continuous variable); the higher the prevalence of TIR5 among FNACs, the higher the operation rate (*p* = 0.029).

**Fig. 4:**
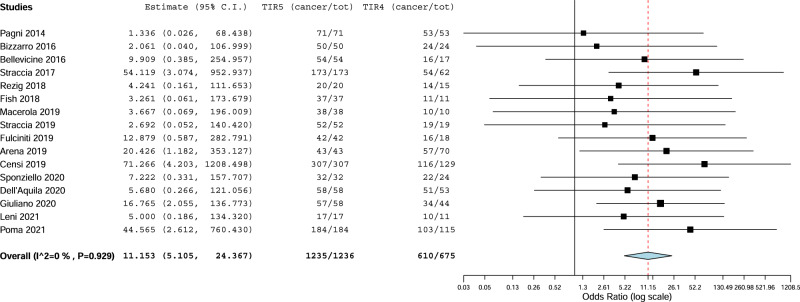
Pooled cancer risk difference between TIR5 and TIR4. Blue diamond indicates the pooled cancer risk difference in the 16 studies and its wideness indicates 95%CI. The cancer risk difference with 95%CI of any study is illustrated by black square and black line, where square size is according to study sample size

Third, the pooled risk difference between TIR5 and TIR4 was calculated. As illustrated in Fig. 4, TIR5 reports was associated with significant cancer risk (OR 11.153) than TIR4. Heterogeneity was absent (I_2_ 0%).

## Discussion

FNAC is pivotal to plan the optimal management of TN patients. In fact, we generally evaluate these patients by ultrasound to select patients eligible to FNAC and, then, we usually recommend surgical treatment when cancer is suspected on cytological preparations. When differentiated thyroid carcinomas are classified at intermediate-to-high risk, international guidelines agree in considering total thyroidectomy with postoperative radioiodine therapy to achieve a complete remission. Facing lower risk patients, experts agree that a less extended approach may be safely managed by more conservative approaches, ranging from total thyroidectomy without radioiodine administration or lobectomy to active surveillance [2, 27]. However, several factors could influence the optimal approach to individual patients, and a proper pre-surgical risk stratification still remains a challenge. Indeed, biopsy cannot assess the histological features consistent with aggressive subtypes of cancer. In addition, while molecular testing has been suggested by some as a possible fix for this issue [2], its efficacy can still be disappointing [28]. To make matters worse, prediction of the FNAC-based risk of malignancy itself can be a challenge. In the three most diffused cytological systems we can find two categories of suspicious for and diagnostic of malignancy, such as V and VI in TBRSTC [4], Thy4 and Thy5 in RCPath [3], and TIR4 and TIR5 of ICCRTC [6]. While categories VI, Thy5, and TIR5 are expected to be associated with a near-to-100% cancer prevalence at histology, the estimated risk of cancer of the classes V, Thy4, and TIR4 may vary between 50 to 75% [4], 68 to 70% [3], and 60 to 80% [6], respectively. Remarkably, these figures were not initially based on specific studies assessing the actual risk of malignancy, but they were estimated by the expert boards when preparing guidelines. The performance of TBRSTC was later evaluated in a systematic review recording a cancer rate of 79.6% in V and 99.1% in VI [29], and that of RCPath in another systematic review which found 79% in Thy4 and 98% in Thy5 [30]. Importantly, the Vuong meta-analysis [29] reported the pooled finding of the operation rate as factor which can have a potential to influence the cancer rate. As for the Italian system, a preliminary meta-analysis on initial data was published [7]. There, the risk of cancer of TIR4 and TIR5 was 85 and 99%, respectively. However, as above mentioned, the number of studies and their sample size were limited, and the operation rate or other influencing factors for both TIR4 and TIR5 cases were not analyzed, being unavailable in the literature. The present systematic review was then conceived to achieve higher-level evidence about the risk of cancer associated with TIR4 and TIR5. The herein adopted criteria to include studies were highly selective. In addition, the resection rate data were extracted from each study and other potential influencing factors were considered. With these premises, the figures obtained in the present meta-analysis have to be regarded as highly reliable and they can form a solid basis upon which Italian guidelines [6] can estimate the risk of cancer associated to TIR4 and TIR5 in an updated version.

First, the herein found cancer rate in TN classified as TIR4 was 92.5%, with a fairly narrow 95%CI and a moderate inconsistency. This figure corroborates the preliminary data [7] and questions the estimates reported in ICCRTC. Second, the pooled cancer rate among TIR5 was 99.7%, without heterogeneity. This finding confirms the preliminary one [7] and makes the original estimates of malignancy reliable. Third, the 95%CI of cancer rate in TIR4 and TIR5 was not overlapping, meaning this that there is a significantly different risk between them. In addition, the cancer risk associated with TIR5 was significantly higher than that of TIR4 with OR 11. These features actually make TIR4 and TIR5 two distinct categories. Fourth, regarding cancer rate findings, heterogeneity was found only in TIR4 and remained not fully explained after several sub-analyses. However, since heterogeneity was found in resection rate in both TIR4 and TIR5, the performed sub-analyses could allow to partially explain the above inconsistency of cancer rate among TIR4. In fact, the mean frequency of TIR4 and TIR5 among all FNACs included in the 16 studies varied significantly (i.e., from 1.1 to 29.6% and 1.9 to 70.4%, respectively). When we analyzed the impact of these frequencies on the resection rate, we found the latter was significantly influenced by the frequency of cases both in TIR4 and in TIR5 (i.e., the higher the prevalence of TIR4/TIR5 among FNACs, the higher their operation rate). This data might suffer from a publication bias. In example, two large series included in our study [13, 14] derive from metropolitan institutions that represent referral centers for thyroid FNAC. In these two studies there was a low operation rate of both TIR4 and TIR5 which may be due to the fact that TN patients, after FNAC, were managed elsewhere and the authors have no follow-up data. These findings mean that several factors could influence the results we read in these papers. In fact, we cannot fully know how each institution manage TN patients, how select them for FNAC, and how and when recommend surgery. In addition, the expertise of local cytopathologist remains not explored, but its influence cannot be excluded [31]. Furthermore, one role may be hold in this context by molecular tests. Some of the included papers [12, 16–19, 21–23, 26] used molecular tests with different combinations (i.e., BRAF as single test, BRAF combined with TERT, or different extended molecular panels). Because of this different approaches, pooled findings could not be calculated. Anyway, as suggested by these studies [16, 17], molecular tests did not increase the diagnostic accuracy of TIR4 and TIR5 categories. Finally, the compliance of each patient and the availability of surgical facilities during pandemic could have had an impact on operation rate [32]. Lastly, the cancer rate herein found in TIR4 (92.5%) seems to be higher than that reported in other meta-analysis in category V of TBRSTC (79.6%) [29], and in Thy4 of RCPath (79%) [30]. This finding merits a careful evaluation by cytopathologists to understand whether it depends on the definition of the classes of suspicious for malignancy or on other factors. One possible explanation of the cancer rate found in TIR4, also higher than that estimated in ICCRTC guidelines [6], might be the introduction in 2014 of two subcategories of TIR3 (i.e. low-risk TIR3A and high-risk TIR3B). In fact, TIR3B “*also includes samples characterized by nuclear alterations suggestive of papillary carcinoma, which do not permit to reliably exclude malignancy, but are too mild or focal to be included in the TIR4 category*” [6]. Then, the pathologists may have been pushed to downgrade in TIR3B some cases that would have been previously classified as TIR4 [5]. This data could be investigated in future studies.

Both limitations and strengths of the present systematic review have to be discussed. First, basically all papers included retrospective series of TN patients managed in several institutions according to local management rules. Some concerns may then be present about selection bias. Second, all studies retrieved with the present systematic were from Italy, as largely expected. Then, these data are reliable as they derive from institutes that use ICCRTC in their clinical routine. Third, the resection rate of both TIR4 and TIR5 was very high. Being this data in line with the indication contained in the ICCRTC guidelines, this can represent a proof of good practice followed in the institutions involved in the 16 studies.

In conclusion, the actual risk of malignancy of TIR4 and TIR5 of ICCRTC is 92.5 and 99.7%, respectively. These figures can form the basis for the next updated version of ICCRTC. Any institution using ICCRTC is asked to revise its series of TIR4/TIR5, calculate their cancer rate among operated cases, and, importantly, consider all the modifiers of the risk of malignancy, e.g., clinical management, percentage of TIR4/TIR5 among the overall series of FNACs, and resection rate. Ideally, a cross-check among institutions should be considered.

## Supplementary Information

Supplemental Table
