# Supplementary material for: Operation rate and cancer prevalence among thyroid nodules with FNAC report of suspicious for malignancy (TIR4) or malignant (TIR5) according to Italian classification system: a systematic review and meta-analysis
Source: Endocrine. 2022 Aug 20;78(1):24–31. doi: 10.1007/s12020-022-03165-x (PMC9474526; doi:10.1007/s12020-022-03165-x)
Supplement: Supplementary file 1 — Supplemental Table [file 12020_2022_3165_MOESM1_ESM.docx]

|  | 1 | 2 | 3 | 4 | 5 | 6 | 7 | 8 | 9 | 10 | 11 | 12 | 13 | 14 | Tot |
| --- | --- | --- | --- | --- | --- | --- | --- | --- | --- | --- | --- | --- | --- | --- | --- |
| Arena 2019 | Yes | Yes | Yes | Yes | No | Yes | Yes | NA | NA | NA | Yes | No | Uncl | NA | 7 |
| Bellevicine 2016 | Yes | Yes | Yes | Yes | No | Yes | Yes | NA | NA | NA | Yes | No | No | NA | 7 |
| Bizzarro 2016 | Yes | Yes | Yes | Yes | No | Yes | Yes | NA | NA | NA | Yes | No | Uncl | NA | 7 |
| Censi 2019 | Yes | Yes | Yes | Uncl | No | Yes | Yes | NA | NA | NA | Yes | No | Yes | NA | 7 |
| Dell’Aquila 2020 | Yes | Yes | Yes | Yes | No | Yes | Yes | NA | NA | NA | Yes | No | Yes | NA | 8 |
| Fish 2018 | Yes | Yes | Yes | Yes | No | Yes | Yes | NA | NA | NA | Yes | No | No | NA | 7 |
| Fulciniti 2019 | Yes | Yes | Uncl | Yes | No | Yes | Yes | NA | NA | NA | Yes | No | No | NA | 6 |
| Giuliano 2020 | Yes | Yes | Uncl | Yes | No | Yes | Yes | NA | NA | NA | Yes | No | Yes | NA | 7 |
| Leni 2021 | Yes | Yes | Yes | Yes | No | Yes | Yes | NA | NA | NA | Yes | No | No | NA | 7 |
| Macerola 2019 | Yes | Yes | Yes | Yes | No | Yes | Yes | NA | NA | NA | Yes | No | No | NA | 7 |
| Pagni 2014 | Yes | Yes | Yes | Yes | No | Yes | Yes | NA | NA | NA | Yes | No | Yes | NA | 8 |
| Poma 2021 | Yes | Yes | Yes | Yes | No | Yes | Yes | NA | NA | NA | Yes | No | No | NA | 7 |
| Rezig 2018 | Yes | No | Uncl | Yes | No | Yes | Yes | NA | NA | NA | Yes | No | Uncl | NA | 5 |
| Sponziello 2020 | Yes | Yes | Uncl | Yes | No | Yes | Yes | NA | NA | NA | Yes | No | No | NA | 6 |
| Straccia 2017 | Yes | Yes | Yes | Yes | No | Yes | Yes | NA | NA | NA | Yes | No | No | NA | 7 |
| Straccia 2019 | Yes | Yes | Uncl | Yes | No | Yes | Yes | NA | NA | NA | Yes | No | Yes | NA | 7 |

**Supplemental Table 1. Quality assessment**

1. Was the research question or objective in this paper clearly stated?

2. Was the study population clearly specified and defined?

3. Was the participation rate of eligible persons at least 50%?

4. Were all the subjects selected or recruited from the same or similar populations (including the same time period)? Were inclusion and exclusion criteria for being in the study prespecified and applied uniformly to all participants?

5. Was a sample size justification, power description, or variance and effect estimates provided?

6. For the analyses in this paper, were the exposure(s) of interest measured prior to the outcome(s) being measured?

7. Was the timeframe sufficient so that one could reasonably expect to see an association between exposure and outcome if it existed?

8. For exposures that can vary in amount or level, did the study examine different levels of the exposure as related to the outcome (e.g., categories of exposure, or exposure measured as continuous variable)?

9. Were the exposure measures (independent variables) clearly defined, valid, reliable, and implemented consistently across all study participants?

10. Was the exposure(s) assessed more than once over time?

11. Were the outcome measures (dependent variables) clearly defined, valid, reliable, and implemented consistently across all study participants?

12. Were the outcome assessors blinded to the exposure status of participants?

13. Was loss to follow-up after baseline 20% or less?

14. Were key potential confounding variables measured and adjusted statistically for their impact on the relationship between exposure(s) and outcome(s)?
